# CDK2-Mediated Phosphorylation of *Bombyx mori* FADD Regulates S-Phase Progression and DNA Replication

**DOI:** 10.34133/research.1441

**Published:** 2026-09-22

**Authors:** Lan-Xing Wang, Yan-Bi Long, Si-Yi Wei, Ming-Li Zhang, Xin-Yue Jia, Zhan-Qi Dong, Cheng Lu, Peng Chen, Min-Hui Pan

**Affiliations:** State Key Laboratory of Resource Insects, Key Laboratory of Sericultural Biology and Genetic Breeding, Ministry of Agriculture and Rural Affairs, Southwest University, Chongqing, China.

## Abstract

As a fundamental life process, the cell cycle supports organismal development and genetic material stability. Phosphorylation is the principal molecular mechanism whereby the cyclin-dependent kinase (CDK)–cyclin complex triggers and regulates key events of the cell cycle, ensuring the temporally ordered progression of the cycle. Herein, *Bombyx mori* FAS-associated death domain (FADD) (BmFADD) was identified as a phosphorylation-dependent cell cycle regulator. We demonstrated that BmCDK2 phosphorylates BmFADD at serine 130 (Ser130), preventing its lysosomal–autophagic degradation and promoting a specific interaction (amino acids 69 to 135). This interaction drives the nuclear translocation of BmFADD and acts as a competitive regulator of BmCyclinA–BmCDK2 binding to prevent premature S-phase exit. Furthermore, we generated a transgenic silkworm strain with silk-gland-specific knockout of the *BmFADD* gene and found that loss of BmFADD activated mTOR, promoting endoreplication in silk gland cells and enhancing silk yield. Collectively, our results revealed a phosphorylation-dependent regulatory axis in which BmCDK2-mediated BmFADD phosphorylation at Ser130 competes against BmCyclinA that orchestrates S-phase progression. We confirmed that BmFADD modulates endoreplication through the mTOR signaling pathway. These findings expand the role of FADD in invertebrates, provide novel insights into posttranslational cell cycle regulation, and identify molecular targets for genetically engineering silkworm silk glands.

## Introduction

The cell cycle is a fundamental life process underpinning organism growth and development. Comprising 4 ordered phases—G1, S, G2, and M—a complete cell cycle ensures faithful genome duplication and division [1,2]. Mitosis represents the primary and most prevalent form of cell division in the cell cycle. In mitosis, cells divide to produce 2 daughter cells after DNA replication and chromosome segregation. In addition to this fundamental mechanism, there are special forms of the cell cycle, such as endoreplication. Endoreplication is a variant cell cycle in which repeated rounds of DNA replication occur without intervening mitosis, leading to polyploidy. By expanding the functional plasticity of organisms, these noncanonical cell cycle forms enable organisms to transcend the constraints of conventional cell division. Additional research is needed to elucidate the regulatory mechanisms involved in the cell cycle.

The orderly progression of the cell cycle is due to cyclins and cyclin-dependent kinases (CDKs), which, through their periodic activity, establish key checkpoints that guard phase transitions [3,4]. Cyclins combine with CDKs to form active holoenzymes, regulating the phosphorylation and dephosphorylation of various period cycle-related proteins, thereby controlling the cell cycle process. Mitogenic signaling triggers G1 entry by inducing CyclinD–CDK4/6 formation, which phosphorylates retinoblastoma to partially activate E2F and initiate CyclinE expression. The subsequent CyclinE–CDK2 activity fully inactivates retinoblastoma, enabling robust E2F activation and S-phase commitment. During the S phase, CyclinA–CDK2 sustains replication, and the later CyclinA–CDK1 and CyclinB–CDK1 complexes drive progression through G2 and into mitosis, respectively, to complete division [5–12]. Orderly progression of the cell cycle is indispensable for normal development and for the formation of organs in various organisms. However, the mechanistic details of this phosphorylation-driven regulatory loop, particularly how it is coordinated with other core cell cycle machinery, remain to be precisely defined.

FAS-associated death domain (FADD) is an evolutionarily conserved cytoplasmic adaptor protein that contains a death domain (DD) [13]. FADD is widely conserved across species and was initially identified as a core adaptor that propagates apoptotic signals from death receptors [14]. Apart from being an important tool for mediating apoptosis, FADD is involved in cell proliferation, cell cycle progression, tumor development, and autophagy [15,16]. Research has revealed that FADD mediates numerous functions independent of death receptors. These nonapoptotic roles are governed by its subcellular localization and posttranslational modifications, particular phosphorylation [17,18]. Different kinases phosphorylate FADD at specific residues, producing a series of cellular effects [19–23]. The identification of critical regulatory kinases mediating FADD phosphorylation in mammals, the functional specificity of individual phosphorylation sites, and the precise regulatory mechanism underlying these events in the cell cycle constitute the main unresolved issues. Research on insect FADD has been confined to its apoptotic and immune functions, while its phosphorylation-mediated nonapoptotic roles and the underlying mechanisms involved remain poorly understood.

In the silkworm (*Bombyx mori*), an economically important silk-producing insect in the order Lepidoptera, silk protein synthesis relies on the efficient endoreplication of silk gland cells. Silk gland cells undergo approximately 10 cell divisions, followed by 17 to 19 rounds of replication without nuclear division or cytoplasmic division, ultimately forming polyploid cells [24,25]. The massive secretion of silk proteins is directly driven by polyploidy in the silk gland, rendering it crucial to define the precise regulation of the endoreplication in silkworm cells. Existing evidence suggests that multiple genes are involved in regulating the endoreplication of silk gland cells in silkworms, such as the transcription factors *BmZFP67*, *BmSu(H)*, *BmBR-C Z2*, and *BmJun*, as well as the cell-cycle-related genes *BmE2F4*, *BmE2F1*, and *Bmcdc25* [26–32]. The core regulatory networks underlying endoreplication remain incompletely understood, and the mechanistic details require further elucidation.

In this study, we investigated the regulatory function and mechanism of the *B. mori FADD* (*BmFADD*) gene in the cell cycle of silkworms. We confirmed that the BmFADD protein participates in S-phase regulation after phosphorylation by BmCDK2 at serine 130 and modulates endoreplication through the mTOR signaling pathway. These findings provide a theoretical foundation for clarifying the regulatory mechanism of phosphorylation modifications during cell cycle progression, offer a perspective for understanding the biological function of BmFADD, and provide molecular targets to advance the genetic engineering of silkworm silk glands.

## Results

### Identification and expression profile of the *BmFADD* gene

**Fig. 1.**
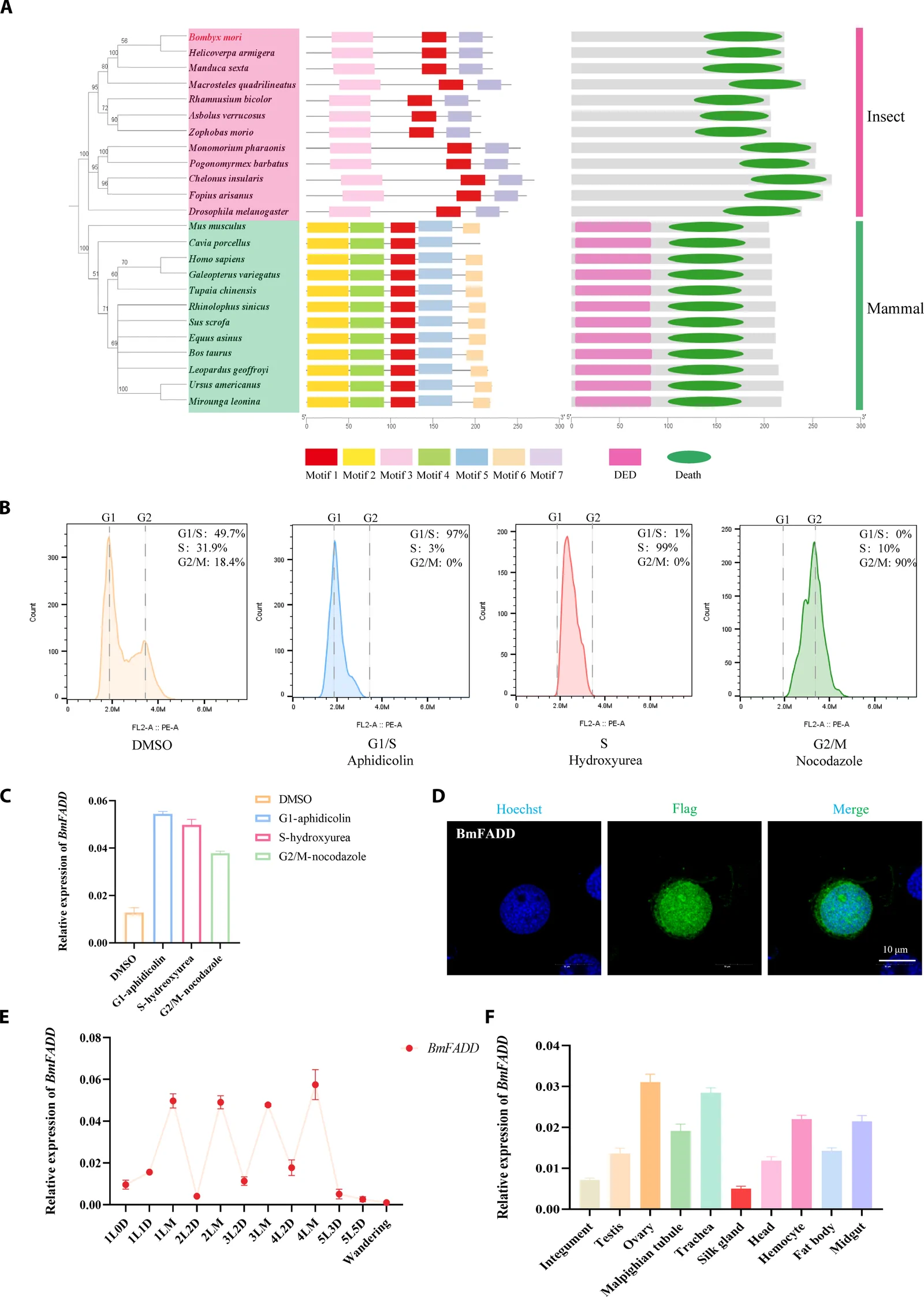
Identification and expression profile analysis of the *Bombyx mori FADD* (*BmFADD*) gene. (A) The phylogenetic tree of FAS-associated death domain (FADD) proteins from insects and mammals include conserved motifs and function domains. (B) Analysis of cell cycle phases via flow cytometry following treatment with cell synchronization agents. (C) Quantitative real-time polymerase chain reaction (qRT-PCR) analysis of the expression levels of *BmFADD* in different cell-cycle-enriched populations. (D) Immunofluorescence analysis of the subcellular localization of the BmFADD protein; scale bar = 10 μm. (E) qRT-PCR analysis of the expression levels of *BmFADD* in silk gland tissue at different developmental stages. (F) qRT-PCR analysis of the expression levels of *BmFADD* in various tissues on the third day of the fifth-instar silkworm larvae. Data are presented as the mean ± SD of 3 independent biological replicates.

We successfully cloned and characterized the silkworm *FADD* homolog, designated as *Bombyx mori FADD* (*BmFADD*). Localized on chromosome 22, the *BmFADD* gene comprises 2 exons and a single intron. Its coding sequence is 666 bp in length, encoding a 221-amino acid protein that contains a DD at amino acid positions 135 to 217 (Fig. S1A and B). Phylogenetic analysis and conserved motif identification showed that insect and mammalian FADD proteins clustered into distinct clades. Both groups of FADD proteins harbored multiple conserved motifs, among which the DD was highly conserved across all species (Fig. 1A). To evaluate the potential involvement of BmFADD in cell cycle regulation, we detected its expression levels across different cell cycle stages. Aphidicolin, hydroxyurea, and nocodazole were applied to synchronize cells at the G1/S, S, and G2/M transition points, respectively. The efficiency of cell cycle synchronization was validated by flow cytometric analysis. *BmFADD* remained highly expressed throughout the G1 and S phases, whereas its expression decreased at the G2/M phase, with an expression pattern comparable to that of *BmCyclinE* (Fig. 1B and C and Fig. S1C and D). The immunofluorescence assay demonstrated that BmFADD was distributed in both the cytoplasm and nucleus of cells, further providing a spatial prerequisite for its regulatory function in the cell cycle (Fig. 1D). Furthermore, we investigated the spatiotemporal expression patterns of the *BmFADD* gene in silkworms, showing that *BmFADD* exhibited the lowest expression in silk gland tissue, with high expression at the dormant stage and lower expression during the feeding period and the late fifth instar stage (Fig. 1E and F). These results indicate that the *BmFADD* gene may be involved in maintaining the cell cycle and the endoreplication of silk glands.

### *BmFADD* regulates cell cycle progression

**Fig. 2.**
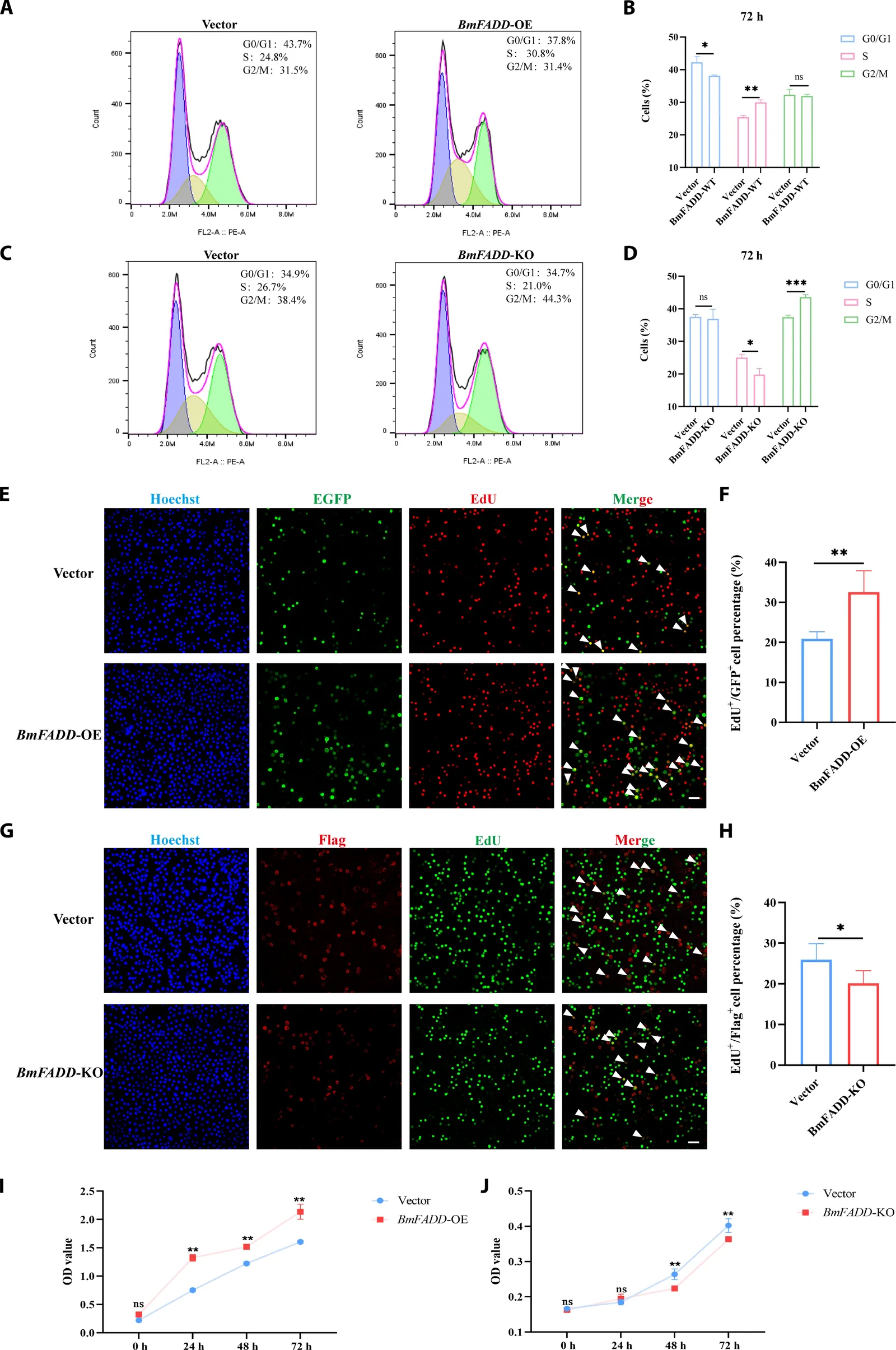
Functional analysis of *Bombyx mori FADD* (*BmFADD*) in the cell cycle and DNA replication. (A and B) Flow cytometry analysis of the effect of *BmFADD* overexpression on cell cycle progression in total cell populations. (C and D) Flow cytometry analysis of the effect of *BmFADD* knockout on cell cycle progression in total cell populations. (E and F) A 5-ethynyl-2′-deoxyuridine (EdU) assay was used to evaluate DNA synthesis following *BmFADD* overexpression; scale bar = 50 μm; *n* = 30; the white arrow represents the positive cells. (G and H) An EdU assay was used to evaluate DNA synthesis following *BmFADD* knockout; scale bar = 50 μm; *n* = 30; the white arrow represents the positive cells. (I and J) Cell Counting Kit-8 (CCK-8) assay of cell viability following *BmFADD* overexpression and *BmFADD* knockout. Data are presented as the mean ± SD of 3 independent biological replicates. ***P < 0.001; **P < 0.01; *P < 0.05; ns, not significant, paired Student *t* test.

To clarify the function of *BmFADD* in the cell cycle of silkworms, we constructed overexpression and knockout vectors (Fig. S2A to D). Following plasmid transfection, cells were collected for subsequent analysis via flow cytometry and 5-ethynyl-2′-deoxyuridine (EdU) labeling experiments. The results showed that *BmFADD* overexpression after 48 and 72 h decreased the proportion of cells in the G1 phase (from 43.7% to 37.8%) and increased the proportion of cells in the S phase (from 24.8% to 30.8%). In contrast, *BmFADD* knockout decreased the proportion of cells in the S phase (from 26.7% to 21.0%) and increased the proportion of cells in the G2/M phase (from 38.4% to 44.3%) (Fig. 2A to D and Fig. S2E). The EdU labeling assay indicated that the proportion of EdU-positive cells in the *BmFADD*-overexpressing group was higher than that in the control group, while the proportion in the *BmFADD*-knockout group was lower (Fig. 2E to H). The results of the Cell Counting Kit-8 (CCK-8) assay confirmed that *BmFADD* overexpression promoted proliferation and *BmFADD* knockout inhibited proliferation (Fig. 2I and J). These results indicate that *BmFADD* positively regulates cell cycle progression by enhanced S phase and DNA replication.

### BmCDK2 mediates BmFADD phosphorylation at serine 130 (Ser130)

**Fig. 3.**
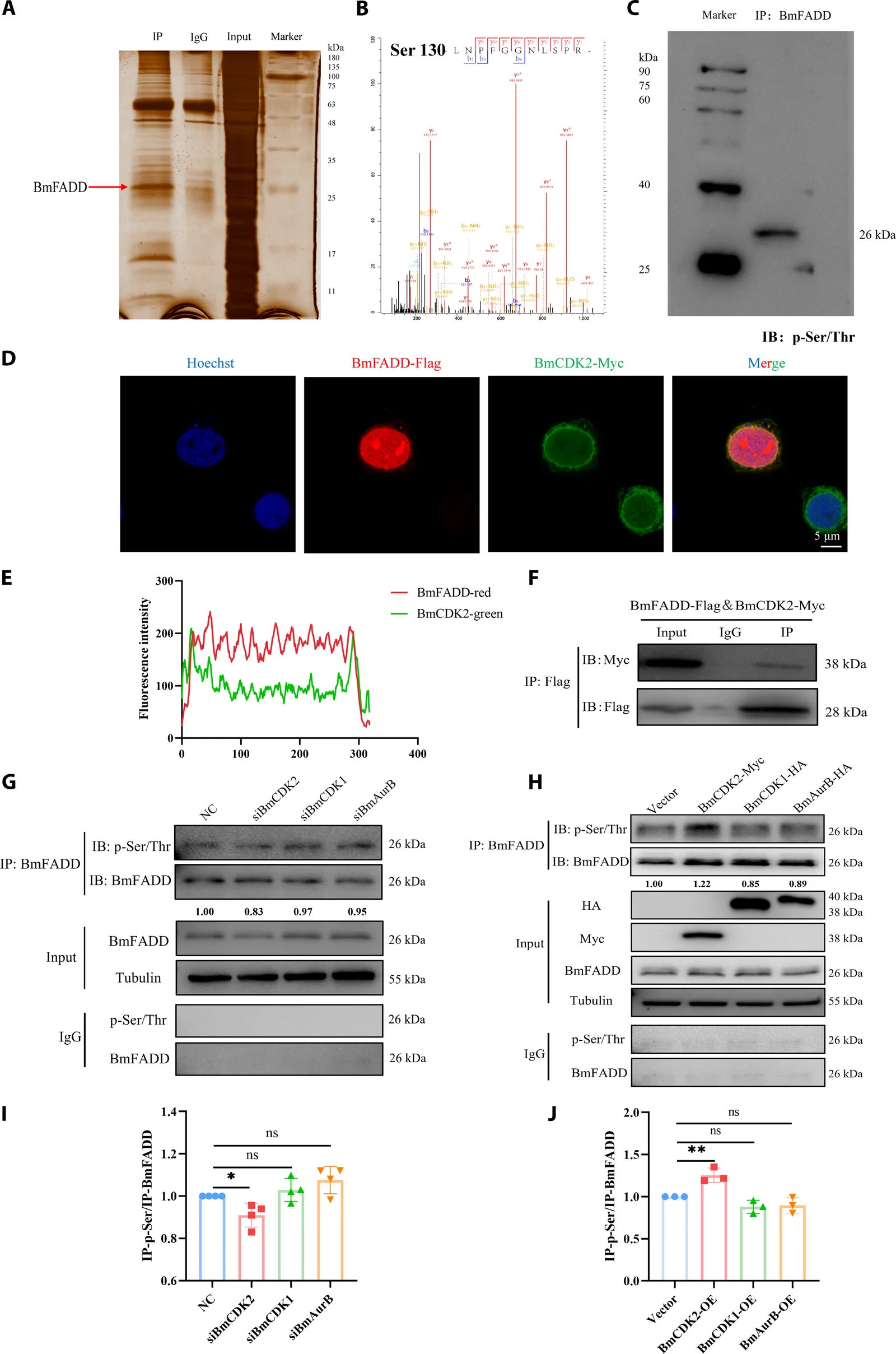
Identification of phosphorylation modification on the *Bombyx mori* FAS-associated death domain (BmFADD) protein. (A) Silver nitrate staining of proteins obtained following BmFADD immunoprecipitation; the red arrow points to the BmFADD protein. (B) Phosphorylated peptides identified via liquid chromatography–tandem mass spectrometry (LC–MS/MS) and Ser130 were phosphorylated. (C) Immunoprecipitation confirmed that BmFADD is subject to serine phosphorylation modification. (D) Immunofluorescence analysis of the colocalization of BmFADD and BmCDK2; scale bar = 5 μm. (E) Analysis of the colocalization distance (pixel) between red and green fluorescence using ImageJ. Pearson’s correlation coefficient = 0.649. (F) Co-immunoprecipitation verification of the interaction between BmFADD and BmCDK2. (G and H) Immunoprecipitation analysis identifying the kinase that modulates BmFADD phosphorylation levels. (I and J) Statistical analysis of the gray value of the BmFADD protein phosphorylation level. Data are presented as the mean ± SD of least 3 independent biological replicates. **P < 0.01; *P < 0.05; ns, not significant, paired Student *t* test.

The above findings highlight the regulatory role of the *BmFADD* gene in cell cycle progression. However, its underlying regulatory mechanism remains to be further elucidated. Studies in mammals have shown that the FADD protein mainly participates in cell cycle regulation via phosphorylation at specific sites. Therefore, we first identified the phosphorylation sites of the BmFADD protein via liquid chromatography–tandem mass spectrometry (LC–MS/MS). The results showed that only Ser130 of BmFADD was phosphorylated. In addition, the immunoprecipitation experiment using endogenous BmFADD antibodies and pan phospho-serine/threonine antibodies confirmed the phosphorylation modification of the BmFADD protein (Fig. 3A to C). Aiming to identify the kinase responsible for phosphorylating BmFADD Ser130, we combined LC–MS/MS analysis with iGPS 1.0 prediction. Our preliminary results suggested that the protein kinases BmCDK1, BmAurB, and BmCDK2 might phosphorylate BmFADD (Fig. S3A and B). Immunofluorescence and co-immunoprecipitation (Co-IP) assays showed that all 3 protein kinases not only colocalized with BmFADD in both nuclear and cytoplasmic compartments but also interacted with BmFADD. However, after knocking down or overexpressing the 3 different kinases, we found that only BmCDK2 affected the phosphorylation level of BmFADD (Fig. 3D to J and Fig. S3C to I). We further investigated the kinase responsible for phosphorylating BmFADD at Ser130 by assessing the phosphorylation level of BmFADD following cotransfection of CDK2 with either wild-type (WT) BmFADD or the Ser130 dephosphorylation mutant (S130A). Phosphorylation was detected only in the WT BmFADD group (Fig. S3J and K). These results suggest that BmCDK2 is the primary kinase mediating BmFADD phosphorylation at Ser130.

### BmCDK2-dependent phosphorylation of BmFADD at Ser130 enhances protein stability

**Fig. 4.**
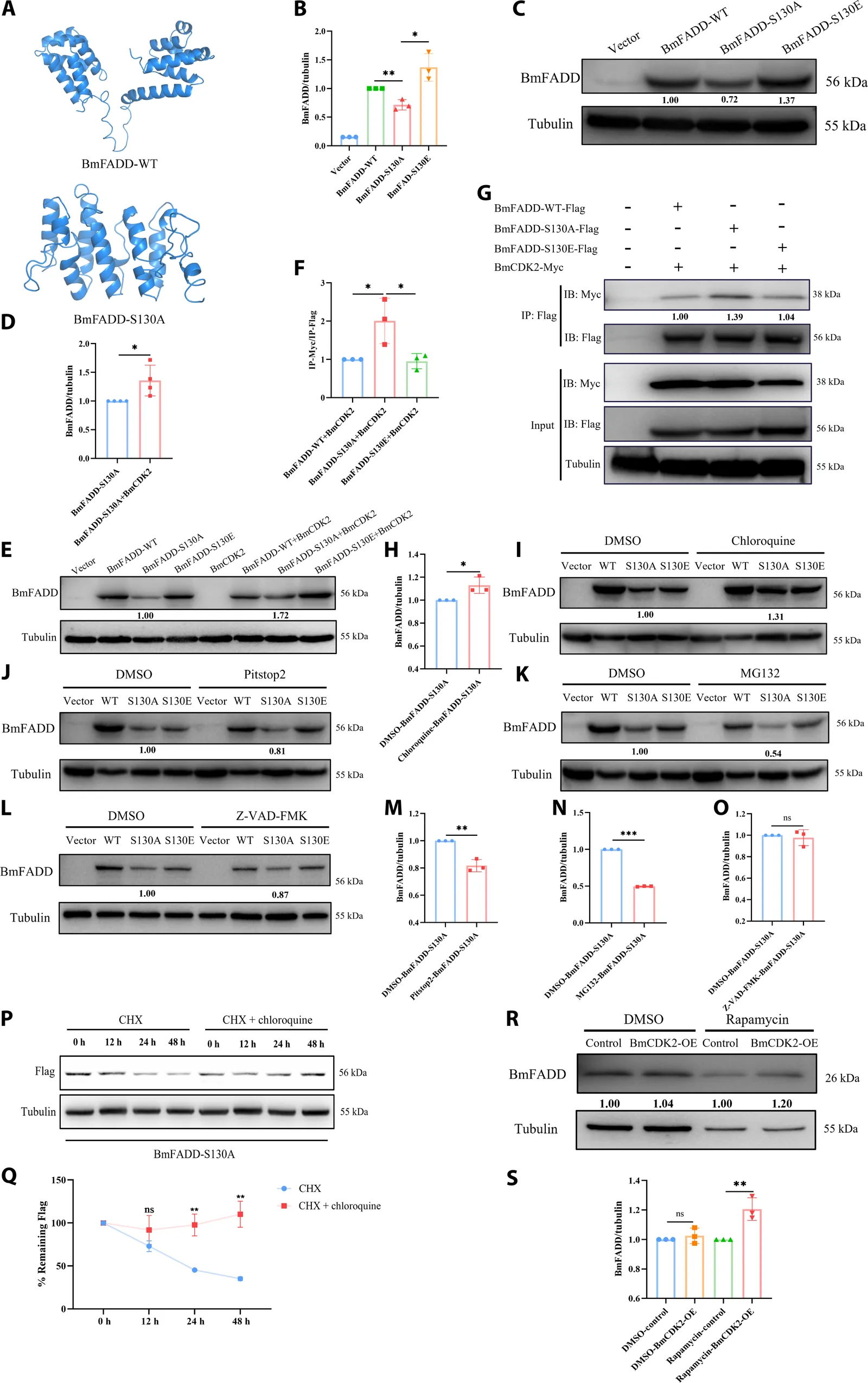
Effect of BmCDK2 on *Bombyx mori* FAS-associated death domain (BmFADD) protein stability. (A) AlphaFold 3 predicted the conformational changes in the BmFADD protein during phosphorylation and dephosphorylation. (B and C) Western blot analysis of the effects of BmFADD protein phosphorylation and dephosphorylation on its stability. (D and E) Western blot analysis of the effect of BmCDK2 on BmFADD stability. (F and G) Co-immunoprecipitation (Co-IP) assay assessing the effects of BmFADD phosphorylation and dephosphorylation on its interaction capability with BmCDK2. (H to O) Western blot assessment of the impact of chloroquine, Pitstop2, MG132, and Z-VAD-FMK on BmFADD stability. (P and Q) Western blot analysis of the effect of cycloheximide (0.3 mg/ml) combined with chloroquine (20 μM) on the degradation of the BmFADD S130A mutant. (R and S) Western blot assessment of the impact of BmCDK2 on the degradation of the BmFADD protein caused by rapamycin. Data are presented as the mean ± SD of least 3 independent biological replicates. ***P < 0.001; **P < 0.01; *P < 0.05; ns, not significant, paired Student *t* test.

Protein stability is an important determinant of protein function. Protein phosphorylation typically functions by altering the protein’s conformation, thereby altering protein stability. Therefore, we investigated the correlation between the phosphorylation of BmFADD Ser130 mediated by BmCDK2 and protein conformation and stability. First, we constructed a dephosphorylation mutant (S130A) and a simulated phosphorylation mutant (S130E) by replacing Ser130 of the BmFADD protein with alanine and glutamic acid, respectively. Subsequently, the 3-dimensional structures of WT and dephosphorylated BmFADD proteins were predicted using the AlphaFold 3 website, and images were generated using the PyMOL software. The results showed that the phosphorylation of Ser130 altered the conformation of the BmFADD protein (Fig. 4A). The western blot analysis further confirmed that the conformational change caused by the phosphorylation of BmFADD Ser130, mediated by BmCDK2, is crucial for maintaining the stability of the protein (Fig. 4B to E). Additionally, we observed that the dephosphorylation mutation of BmFADD Ser130 enhanced the interaction with BmCDK2 (Fig. 4F and G). These findings indicate that BmCDK2-mediated phosphorylation of BmFADD at Ser130 alters its protein conformation, thereby modulating protein stability.

The primary protein degradation pathways in eukaryotes include the ubiquitin–proteasome, lysosome, and the caspase pathways [33]. The BmFADD phosphorylation-mediated protein degradation pathway was evaluated by subjecting cells to 4 specific inhibitors in the degradation pathways: MG132 (ubiquitin–proteasome), Pitstop2 (endocytosis), chloroquine (lysosomal–autophagy), and Z-VAD-FMK (caspase). The western blot results showed that only the lysosomal–autophagy inhibitor (chloroquine) could rescue BmFADD protein degradation caused by its Ser130 dephosphorylation mutation after treatment; the other 3 inhibitors had no such effect. We performed a cycloheximide chase experiment in the presence of chloroquine and confirmed that chloroquine prevented the degradation of the BmFADD S130A mutant. Additionally, protein degradation induced by treatment with the autophagy activator rapamycin could be prevented by BmCDK2 overexpression (Fig. 4H to S and Fig. S4). These results demonstrate that BmCDK2-mediated phosphorylation induces a conformational change in BmFADD, enhances its stability, and rescues BmFADD from degradation via the lysosomal–autophagy pathway.

### BmCDK2 determines the nuclear transport of BmFADD

**Fig. 5.**
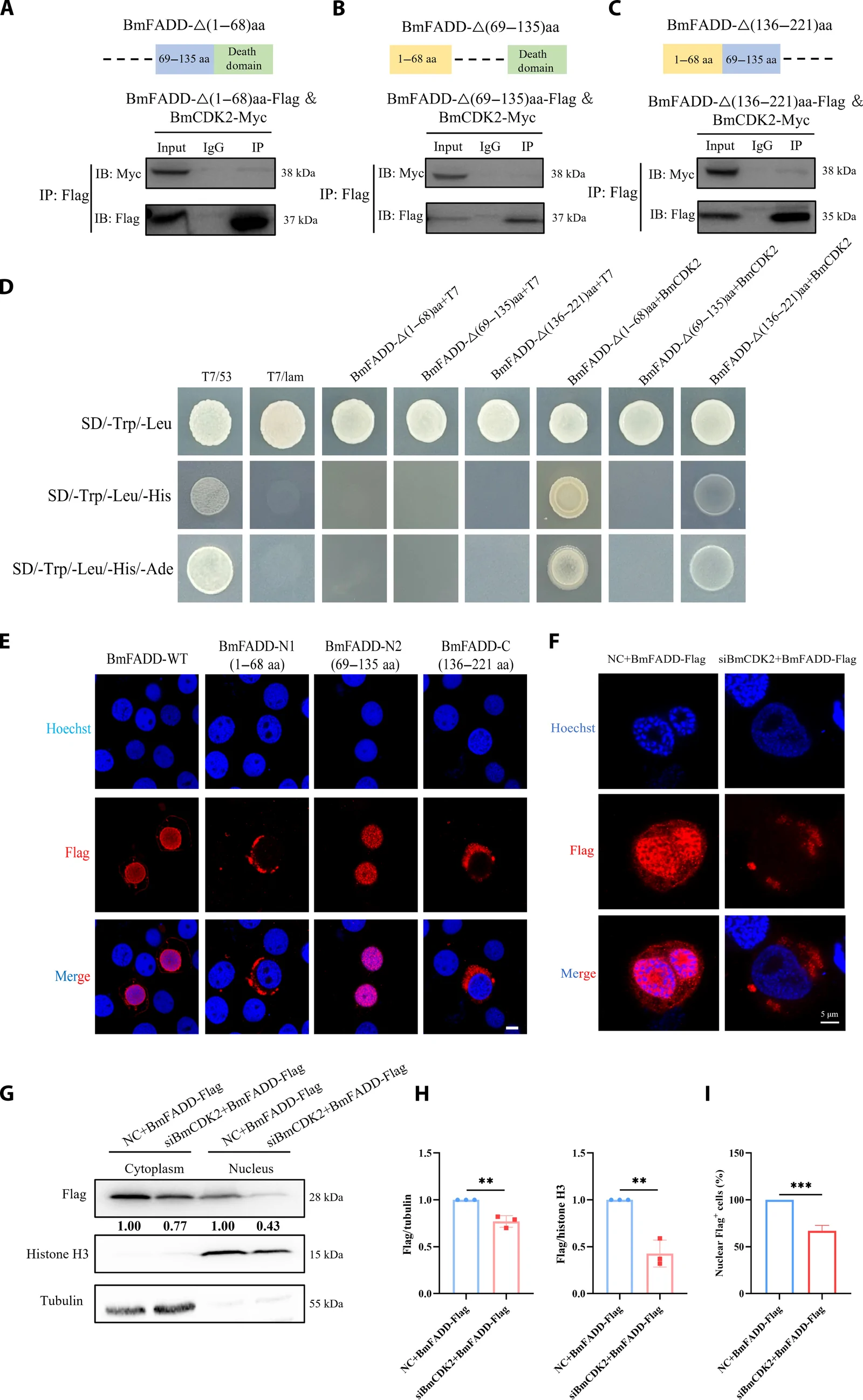
Effect of BmCDK2 on the nuclear localization of the *Bombyx mori* FAS-associated death domain (BmFADD) protein. (A to C) Co-immunoprecipitation (Co-IP) verification of the interaction between BmCDK2 and the truncated mutant of BmFADD. (D) Yeast 2-hybrid system verification of the interaction between BmCDK2 and the truncated mutant of BmFADD. (E) Immunofluorescence analysis of the subcellular localization of various truncated mutants of BmFADD; scale bar = 5 μm. (F) Immunofluorescence analysis of the impact of interfering with BmCDK2 expression on BmFADD subcellular localization; scale bar = 5 μm. (G) Western blot analysis of the BmFADD protein level in the cytoplasm and nucleus after interfering with BmCDK2. (H) Statistical analysis of the gray values of the BmFADD protein in the cytoplasm and nucleus after interfering with BmCDK2. (I) Statistical analysis of the percentage of nuclear-localized Flag^+^ cells. Data are presented as the mean ± SD of 3 independent biological replicates. ***P < 0.001; **P < 0.01, paired Student *t* test.

The results in Fig. 4G showed that Ser130 does not appear to be the key site mediating the interaction between BmCDK2 and BmFADD. Hence, to determine the region where BmCDK2 interacts with BmFADD proteins, we constructed 3 truncated mutants of BmFADD and cotransfected them with BmCDK2 to conduct immunoprecipitation experiments and yeast 2-hybrid (Y2H) assays. The Co-IP and Y2H results showed that BmFADD lacking the 69- to 135-amino acid region could not interact with BmCDK2, and the 69- to 135-amino acid region (the BmCDK2-binding region) was capable of nuclear expression (Fig. 5A to E). Additionally, Ser130 phosphorylation did not affect the nuclear transport of BmFADD (Fig. S5B). As a result, we determined whether BmCDK2 could mediate the nuclear transport of BmFADD protein. Immunofluorescence showed that after interfering with BmCDK2, the BmFADD protein was located in the cytoplasm. Western blot further confirmed that upon interference with BmCDK2 expression, the nuclear import of BmFADD was markedly inhibited (Fig. 5F to I). These results indicate that BmCDK2 interacts with BmFADD amino acids 69 to 135 and promotes the translocation of the BmFADD protein from the cytoplasm to the nucleus.

### BmFADD phosphorylation orchestrates S-phase progression by modulating BmCDK2–BmCyclinA complex formation

**Fig. 6.**
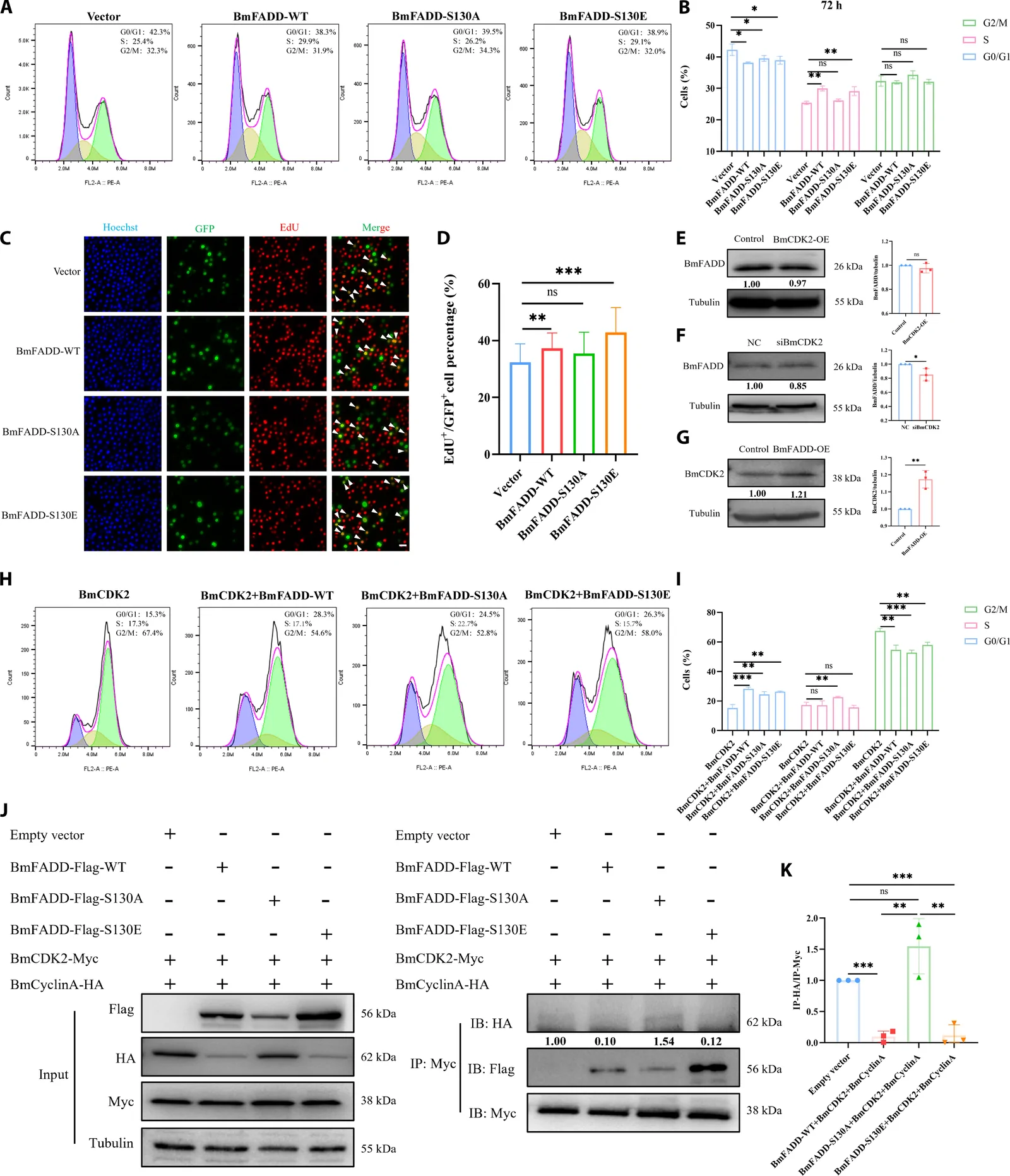
Regulatory mechanism analysis of *Bombyx mori* FAS-associated death domain (BmFADD) protein phosphorylation in cell cycle progression. (A) Flow cytometric analysis of the effects of Ser130 phosphorylation and dephosphorylation on cell cycle progression in total cell populations. (B) Statistical analysis of the percentage of cells in each group. (C and D) A 5-ethynyl-2′-deoxyuridine (EdU) labeling assay was utilized to analyze the effects of Ser130 phosphorylation and dephosphorylation on DNA replication; scale bar = 50 μm; *n* = 30; the white arrow represents the positive cells. (E) Impact of BmCDK2 overexpression on BmFADD expression. (F) The impact of BmCDK2 interference on BmFADD expression. (G) Impact of BmFADD overexpression on BmCDK2 expression. (H and I) Flow cytometric analysis of the impact of BmFADD Ser130 phosphorylation and dephosphorylation on the cell cycle, in conjunction with BmCDK2. (J) Co-immunoprecipitation detection of the interaction capacity between BmFADD Ser130 mutants, BmCDK2, and BmCyclinA. (K) Statistical analysis of the gray value in the immunoprecipitation group of BmFADD Ser130 mutants and BmCDK2 and BmCyclinA proteins. Data are presented as the mean ± SD of least 3 independent biological replicates. ***P < 0.001; **P < 0.01; *P < 0.05; ns, not significant, paired Student *t* test.

To determine whether BmFADD is involved in cell cycle regulation via phosphorylation, cells were transfected with empty vector, WT plasmid, dephosphorylated plasmid (S130A), and simulated phosphorylated plasmid (S130E) for 72 h, after which samples were collected for flow cytometric analysis and EdU labeling assays. Flow cytometric analysis showed that the number of S-phase cells remained relatively unchanged following dephosphorylation. Nevertheless, the number of S-phase cells in the WT and S130E groups increased markedly at both 48 and 72 h after transfection (Fig. 6A and B and Fig. S6A). The EdU labeling experiment also showed no difference in the number of positive cells between the S130A and control groups. However, there were more positive cells in the WT and S130E groups than in the S130A and control groups (Fig. 6C and D). These results demonstrate that BmFADD phosphorylation at Ser130 is involved in S-phase cell cycle regulation.

To explore the molecular mechanism by which BmFADD phosphorylation regulates S-phase progression, we first investigated the regulatory relationship between BmCDK2 and BmFADD. The quantitative real-time polymerase chain reaction (qRT-PCR) and western blot results indicated a reciprocal regulatory relationship between BmFADD and BmCDK2. BmCDK2 knockdown altered the BmFADD protein level, consistent with a kinase–substrate regulatory relationship. Conversely, BmFADD overexpression increased in BmCDK2 expression and BmFADD knockout decreased in BmCDK2 expression (Fig. 6E to G and Fig. S6B and I to K). Flow cytometry showed that the S130A mutant of BmFADD accelerated S-phase progression under BmCDK2 overexpression. In contrast, the WT and S130E groups did not exhibit this effect (Fig. 6H and I). Co-IP further confirmed that phosphorylated BmFADD competes with BmCyclinA for BmCDK2 binding, but not with BmCyclinE (Fig. 6J and K and Fig. S6G, H, L, and M). These results indicate that BmFADD Ser130 phosphorylation modulates S-phase progression by regulating BmCDK2–BmCyclinA complex formation.

### Silk-gland-specific knockout of *BmFADD* promotes endoreplication and silk yield by the mTOR signaling pathway

**Fig. 7.**
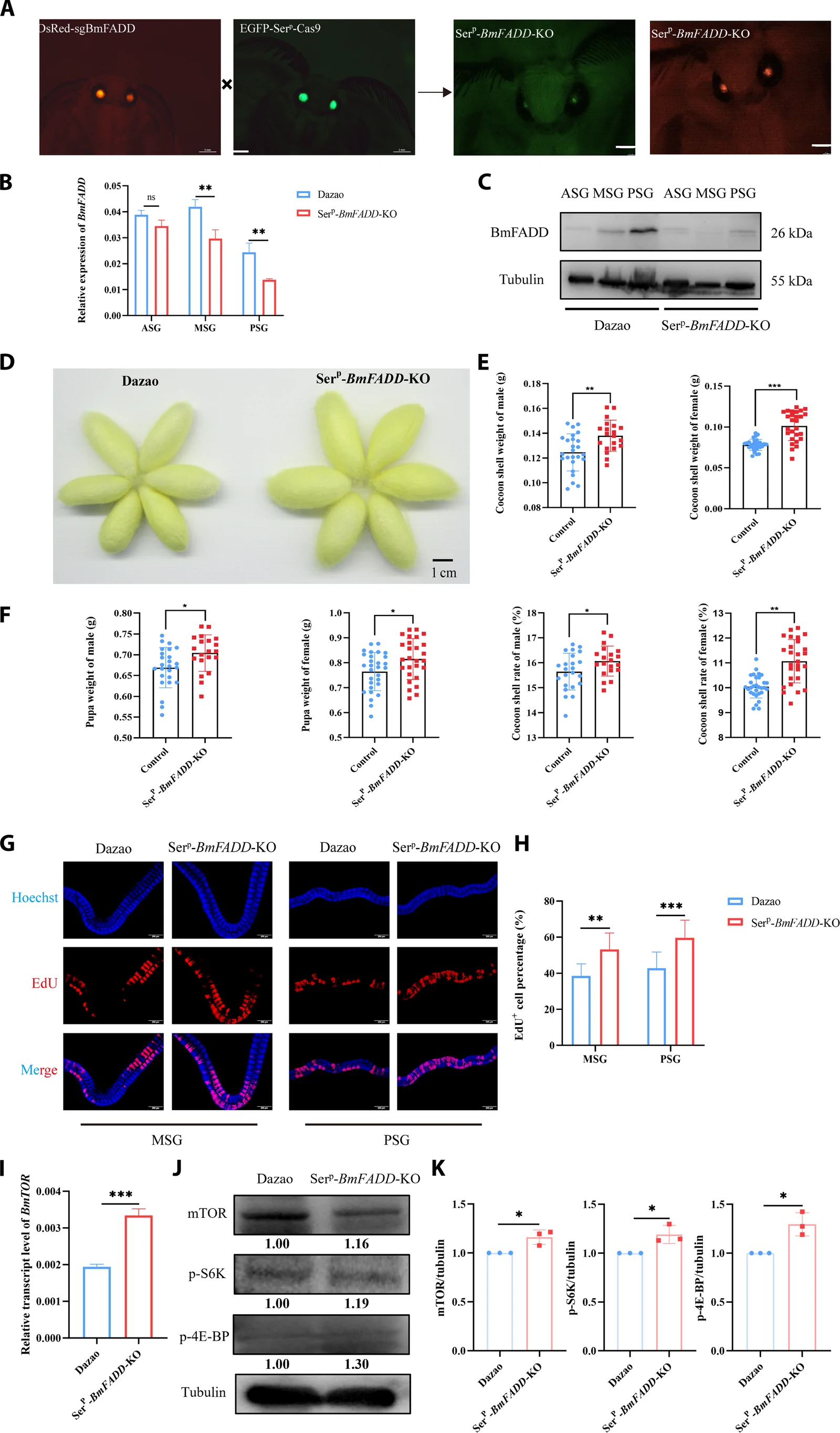
Mechanism analysis of silk production and DNA synthesis in Ser^p^-*BmFADD*-KO strains. (A) Creation of the Ser^p^-BmFADD-KO strain; scale bar = 2 mm. (B) Quantitative real-time polymerase chain reaction (qRT-PCR) analysis of the messenger RNA (mRNA) level of *BmFADD* in the anterior silk gland (ASG), middle silk gland (MSG), and posterior silk gland (PSG). (C) Western blot detection of the protein level of BmFADD in the ASG, MSG, and PSG. (D and E) Analysis of cocoon shell weight; scale bar = 1 cm. (F) Statistical analysis of pupa weight and cocoon shell rate. (G) A 5-ethynyl-2′-deoxyuridine (EdU) labeling assay was performed to evaluate DNA replication in the MSG and PSG; scale bar = 200 μm. (H) Statistical analysis of the percentage of positive cells in the MSG and PSG. (I) qRT-PCR analysis of the mRNA level of *BmTOR* in the silk gland of Dazao and Ser^p^-*BmFADD*-KO strains. (J) Western blot detection of the protein levels of BmTOR, p-4E-BP, and p-S6K in the silk glands of Dazao and Ser^p^-*BmFADD*-KO strains. (K) Statistical analysis of the gray value of BmTOR, p-4E-BP, and p-S6K in the silk gland of Dazao and Ser^p^-*BmFADD*-KO strains. Data are presented as the mean ± SD of least 3 independent biological replicates. ***P < 0.001; **P < 0.01; *P < 0.05; ns, not significant, paired Student *t* test.

To investigate the function of the *BmFADD* gene in the endoreplication of silkworm silk gland cells, we generated silk-gland-specific *BmFADD*-knockout transgenic strains using the CRISPR/Cas9 system and subsequently successfully identified positive transgenic individuals exhibiting both green and red eye fluorescence (Fig. 7A to C). The single-guide RNA (sgRNA)-containing donor construct was integrated between *KWMTBOMO02268* and *KWMTBOMO02269* locating chromosome 4, without affecting the expression of each gene. We further validated the knockout efficiency of *BmFADD* specifically in the silk gland tissue of the resulting transgenic strains. Subsequent analysis confirmed that the *BmFADD* gene was effectively genetically edited in the silk gland of these transgenic strains, with a knockout efficiency of 50% (Fig. S7A to C). We further assessed the economic traits of the *BmFADD*-knockout strains and found that the cocoon size was larger than that of the control group. Meanwhile, the cocoon shell weight, pupa weight, and cocoon shell ratio were all markedly higher in both male and female individuals of the knockout strains than those in the control group (Fig. 7D to F). Next, we performed an EdU labeling assay to investigate whether the enhanced economic traits of the *BmFADD*-knockout strains were attributable to increased DNA synthesis capacity in silk gland cells. The results showed that, compared with the control group, *BmFADD* knockout increased the proportion of EdU-positive cells in the silk glands of silkworms on the second day of the fourth instar (4L2D) (Fig. 7G and H). These results indicate that the *BmFADD* gene plays a regulatory role in endoreplication of silkworm silk gland cells and that BmFADD knockout markedly enhances the DNA synthesis capacity of silk gland cells, thereby improving silk yield.

In the endoreplicating silk gland tissue, BmFADD exhibited biological function distinct from that observed in mitotic cells. To further elucidate the mechanism by which BmFADD regulates endoreplication, we performed a proteomic analysis to determine effector proteins. Using fold change > 1.2 and *P* value < 0.05 as cutoff thresholds, we identified 170 up-regulated and 138 down-regulated proteins in the transgenic lines. Subsequent EggNOG functional enrichment analysis of these differentially expressed proteins demonstrated that mTOR, a critical regulator of cell cycle progression, was markedly up-regulated in transgenic lines (Fig. S7D and E). qRT-PCR and western blot confirmed that BmFADD knockout enhanced mTOR protein expression. We next examined the expression of key downstream targets of the mTOR pathway, 4E-BP1 and S6K. The results showed that the up-regulated mTOR expression increased the phosphorylation levels of 4E-BP1 and S6K, thereby promoting endoreplication in silk gland cells (Fig. 7I to K). These results demonstrate that BmFADD knockout activates the mTOR signaling pathway, promoting endoreplication in silk gland cells.

## Discussion

*FADD* is a classic gene mediating apoptosis via the death receptor pathway. However, increasing evidence indicates that *FADD* has nonapoptotic roles independent of death receptors in cell proliferation, cell cycle, and tumor development. Research on FADD in insects has mainly focused on apoptosis and innate immunity [34–36]. The nonapoptotic function of the FADD protein in insects is unclear. In this study, we revealed that the silkworm FADD protein is a novel phosphorylation-modulation-dependent cell cycle regulatory factor.

Studies in mammals have revealed that the FADD protein, which is phosphorylated at multiple amino acid sites in its C-terminal region, exerts different biological functions [37]. Nevertheless, we only identified phosphorylation of Ser130 in the homologous FADD protein of the silkworm (Fig. 3A to C). In mouse T cells, phosphorylation of serine at position 191 of the FADD protein (homologous to serine 194 in humans) is a key site involved in cell cycle regulation [38,39]. We found that the simulated phosphorylation mutant BmFADD protein (with glutamate replacing serine) increases the number of cells in the S phase (Fig. 6A and B). Although the Ser130 residues in silkworm proteins differ from those in mammals (Ser191/194) by not showing conservation in the amino acid sequence, they are functionally conserved. It is worth noting that mammalian Ser191/194 is also considered a key site for mediating nuclear translocation. However, our research results showed that Ser130 did not mediate nuclear transport of the BmFADD protein, and the BmFADD sequence did not have a nuclear localization signal (Fig. S5A and B). This offers an opportunity to explore the nuclear entry mechanism of the BmFADD protein. Our research results indicated that the 69- to 135-amino acid region of BmFADD is the key area for binding to BmCDK2 and nuclear import (Fig. 5). Future work may integrate AlphaFold-derived structural models with molecular docking to pinpoint the specific amino acid residues responsible for BmFADD–BmCDK2 binding.

Identified protein kinases for phosphorylated mammalian FADD protein include FIST/HIPK3, CKIα, PLK1, CK2, and AURA [19–23]. Our research provides evidence that CDK2 is the protein kinase responsible for phosphorylating Ser130 of the FADD protein in the silkworm (Fig. 3D to H and Fig. S3J and K). BmCDK2 phosphorylates BmFADD, which has a crucial role in maintaining protein stability. The E3 ubiquitin ligase SPOP in mammals mediates the degradation of the FADD protein [40]. Our findings revealed that BmFADD is degraded via the lysosomal–autophagy pathway rather than the ubiquitin–proteasome pathway. This provides a new molecular basis for understanding the phosphorylation and dephosphorylation regulatory axis of insect FADD protein (Fig. 4).

Many genes are known to be involved in modulating CDK2 activity and the CDK2–cyclin complex [41,42]. We also discovered that not only does BmFADD serve as a substrate for BmCDK2 but it can also regulate the function of BmCDK2 in a feedback manner. Our results indicated that *BmFADD* promotes *BmCDK2* and *BmCyclinE* expression, which was associated with enhanced G1/S transition. *BmFADD* knockout did not alter *BmCyclinE* expression but decreased the expression of *BmCDK2*, further indicating that the effect of BmFADD on S-phase entry was attributed to the BmCDK2–BmCyclinE complex. Consistent with this result, BmFADD did not directly interact with BmCyclinE, suggesting that the increase in BmCyclinE following BmFADD overexpression may represent an indirect response rather than a primary molecular mechanism. In contrast, *BmFADD* knockout increased *BmCyclinA* transcription, suggesting that a compensatory increase in *BmCyclinA* due to the reduction of *BmCDK2* caused by loss of *BmFADD*. Although BmFADD did not directly interact with BmCyclinA, our results indicate that phosphorylated BmFADD competes with BmCyclinA for BmCDK2 binding in the S phase, thus inhibiting S-phase exit (Fig. 6H to K and Fig. S6B to M). Through a phosphorylation-dependent molecular switch, BmFADD serves as a critical substrate and effector for BmCDK2, contributing to the maintenance of appropriate S-phase progression and DNA replication. We also noted that this regulatory effect is limited. When BmCDK2 is excessively overexpressed and cell cycle progression is strongly accelerated, the physiological level of phosphorylated BmFADD may no longer be sufficient to counteract the excessive cell cycle progression. Taken together, BmFADD regulates DNA replication through a complex regulatory network involving multiple mechanisms rather than a single regulatory pathway. Nevertheless, the precise regulatory cascade underlying the BmFADD-dependent modulation of BmCyclinE and the relationship between BmFADD, BmCDK2, and BmCyclinA require further investigation.

Multiple evidence indicates that the function of FADD is highly dependent on cellular and tissue context. In Jurkat cells, overexpression of phosphorylated FADD increases the activity of the nuclear factor-kappa B pathway and alters the expression of cell cycle regulatory proteins, thereby mediating the G2/M transition in the tumor cell cycle [43]. Inducing phosphorylated FADD in prostate epithelial cells leads to cell cycle block at the G2/M phase, preventing uncontrolled proliferation. The degree of dephosphorylation of FADD is greater in prostate cancer cells than in normal epithelial cells. Dephosphorylated FADD promotes the cell cycle progression of prostate cancer cells [44,45]. These findings indicate that the functional consequences of FADD phosphorylation are not uniform across cell types and may depend on the specific cell cycle regulatory environment. Our results also support a tissue-dependent function of BmFADD in *B. mori*. In the silkworm ovarian cell line, which undergoes mitotic cell cycles, BmFADD promoted the G1/S transition, whereas phosphorylated BmFADD further regulates S-phase progression through its interaction with CDK2. In contrast, BmFADD knockout in silk gland cells, which undergo repeated rounds of DNA replication without mitosis, markedly increased EdU incorporation and enhanced DNA synthesis (Fig. 7D to H). In mitotic cells, BmFADD contributes to appropriate S-phase progression, whereas in endoreplicating silk gland cells, it may act as a limiting factor that restrains excessive genome amplification. Thus, BmFADD does not appear to function as a positive or negative regulator of DNA replication; rather, its effect depends on the cellular context and the mode of cell cycle progression. Further studies are needed to clarify the basis for these differences.

The mTOR signaling pathway serves as a central regulator of cell growth, metabolism, and survival. Previous studies have demonstrated that this pathway plays a pivotal role in driving endoreplication in *Drosophila* salivary glands, prothoracic glands, and *Bombyx* silk glands [46–51]. Our findings revealed that BmFADD modulates endoreplication in silkworm silk gland cells through the mTOR signaling pathway, although the precise regulatory mechanisms remain to be further elucidated (Fig. 7I to K). The proteomic analysis showed alterations in some upstream regulators of mTOR, including Ras and SLC family proteins, suggesting that BmFADD may not directly modulate mTOR activity. These findings advance the understanding of BmFADD’s function in cell cycle progression and endoreplication in silkworm cells. Further studies are required to determine how BmFADD is integrated into the distinct CDK- and mTOR-dependent regulatory networks governing mitotic proliferation and endoreplication.

**Fig. 8.**
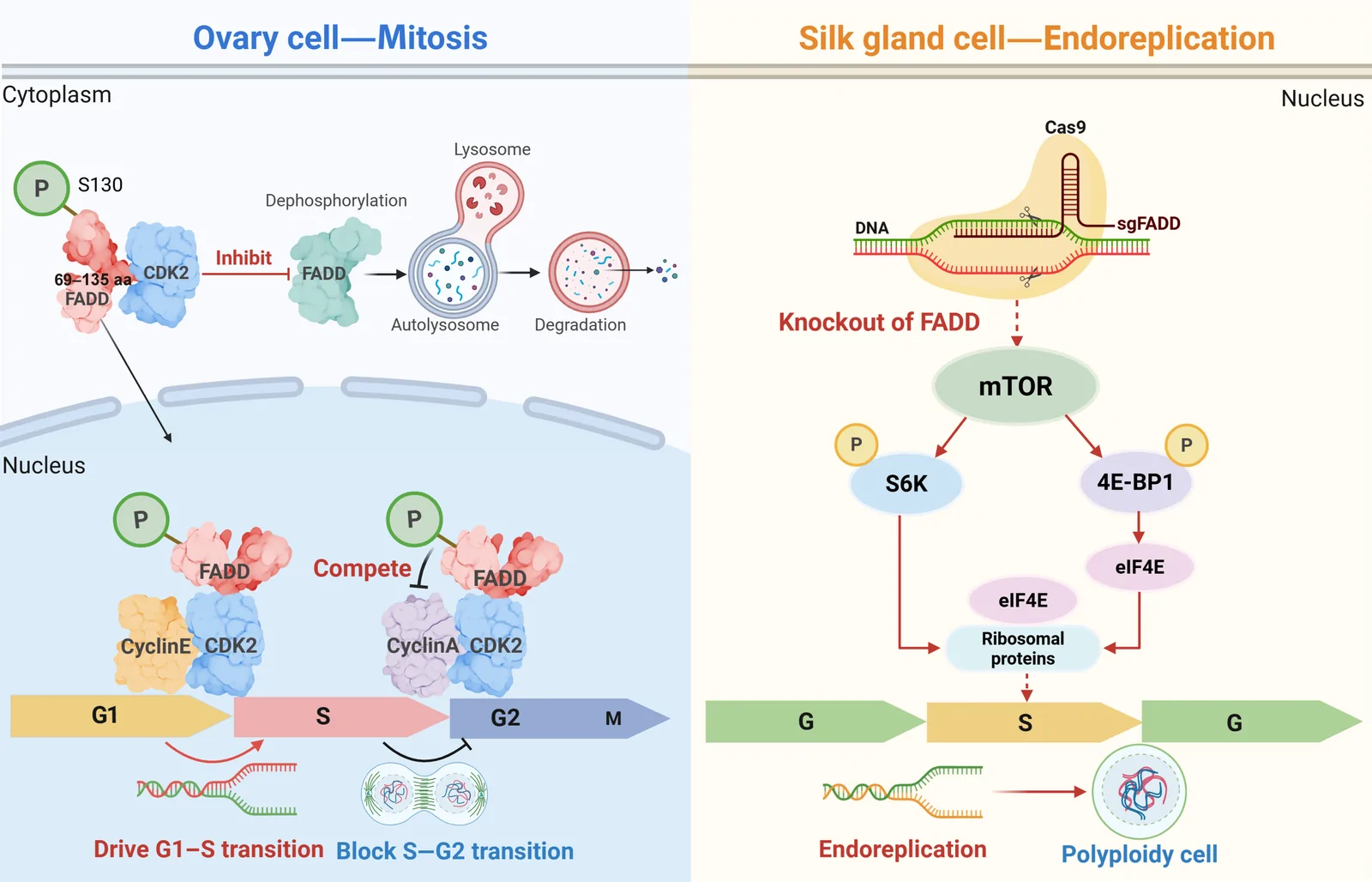
Schematic diagram of the regulatory mechanisms in the mitosis and endoreplication of *Bombyx mori* FAS-associated death domain (BmFADD).

In conclusion, our findings indicate that the BmFADD protein, a cell cycle regulatory factor in silkworms, functions via phosphorylation modification. BmCDK2 binds to the 69- to 135-amino acid region of BmFADD protein and mediates the nuclear transport of this protein and the phosphorylation of serine 130, thus maintaining the stability of the protein and avoiding degradation through the lysosomal–autophagy pathway. The stabilized BmFADD positively regulates CDK2 expression to facilitate G1/S transition and competitively inhibits with CyclinA binding to CDK2, thereby preventing premature S-phase exit. In silk gland cells, BmFADD regulates endoreplication through the mTOR signaling pathway (Fig. 8). These findings establish a new theoretical framework for understanding the cell cycle and endoreplication in silkworms. Future studies should focus on elucidating the role of BmFADD in regulating endoreplication within silkworm silk gland cells.

## Materials and Methods

### Cell line and silkworm strain

The BmN cell line (silkworm ovary) [52] was cultured in TC-100 medium (US Biological) supplemented with 10% fetal bovine serum (BioInd) at 27 °C. The silkworm strain was fed fresh mulberry leaves and housed under a 12-h light/12-h dark photoperiod at 25 °C.

### Bioinformatics analysis

Gene sequence information was sourced from the National Center for Biotechnology Information website. The phylogenetic tree of FADD was generated using MEGA 6.0 with the neighbor-joining method. Conserved motifs were identified using the MEME website. The InterPro website was used to develop the function domain. The phylogenetic tree with motifs and function domains was drawn in TBtools-II [53]. The prediction of nuclear localization signals was carried out using the NLStradamus website. iGPS 1.0 was used to predict phosphorylation modification sites and kinases. Protein structure prediction was conducted using AlphaFold 3. Immunofluorescence colocalization analysis was performed using ImageJ.

### Vector construction and transient transfection

The *BmFADD* gene was inserted into the pIZ/V5 vector to construct the overexpression plasmid. The sgRNA designed using the CRISPR direct website was ligated into the pSL1180-IE1-Cas9-U6 vector to generate the knockout plasmid. The primers used in this study are provided in Table S1. TransIT-Insect Transfection Reagent (Mirus) was used to deliver plasmids into cells, and the transfection system was maintained for 72 h.

### RNA extraction and qRT-PCR

RNA was isolated using an Omega Bio-Tek kit (Norcross, USA), followed by complementary DNA synthesis using the reagents supplied by Accurate Biology (Hunan, China). SYBR qPCR SuperMix Plus (Novoprotein, Shanghai, China) was used for qRT-PCR, and the assay was performed on a real-time PCR system (Bio-Rad, CA, USA). The silkworm *eukaryotic translation initiation factor 4A* gene (*sw22934*) was used as an internal control. The primers are listed in Table S1.

### CCK-8 assay

Cell suspensions (100 μl) from different treatment groups were added to each well of a 96-well plate. CCK-8 solution (10 μl; Yeasen Biotechnology, Shanghai, China) was added to the wells, and the culture plate was incubated at 37 °C for 2 h. The optical density value was measured at 540 nm.

### Flow cytometry analysis

Following transfection of BmN-SWU1 cells with different plasmids for 72 h, the cells were collected and fixed overnight fixation in 70% ethanol at −20 °C. In accordance with the kit protocol (Beyotime, Shanghai, China), cells were incubated with a staining solution containing RNase A and propidium iodide at 37 °C for 30 min. The experimental data were recorded using flow cytometry (CytoFLEX, Beckman Coulter, CA, USA).

### Cell synchronization

Cells from the same logarithmic-phase culture were arrested at the G1/S boundary through treatment with 5 mg/ml aphidicolin (Sigma, Missouri, USA) for 24 h. Synchronization at the G2/M boundary was achieved via 24-h treatment with 10 mg/ml nocodazole (Sigma). For S-phase cell enrichment, cells were exposed to 0.8 mg/ml hydroxyurea (Sigma) for 16 h, followed by the removal of hydroxyurea and a 7-h period of additional culture. Cells isolated from different cell cycle phases were subjected to flow cytometry to assess their corresponding cell cycle stages.

### Immunofluorescence and EdU assay

After transfection with different vectors, cells were cultured for 72 h and then incubated with the EdU reagent (Beyotime) for 2 h. Cells were treated with 4% paraformaldehyde (Beyotime) and 1% Triton X-100 successively for 15 min at 25 °C and then incubated with the primary antibodies, anti-Myc (1:200; Bioworld, Minnesota, USA), anti-FLAG (1:200; Abmart, Shanghai, China), and the secondary antibodies, Alexa Fluor 488 (1:500; Thermo Fisher Scientific, Waltham, MA, USA) and Alexa Fluor 555 (1:500; Thermo Fisher Scientific), for 1.5 h at 37 °C. Images were captured with an FV3000 confocal microscope (Olympus, Tokyo, Japan).

### Construction of the transgenic strain

The sgRNA that exhibited the highest knockout efficiency against BmFADD was selected and cloned into a piggyBac vector. Eggs collected within 6 h of laying were microinjected with the plasmid, sealed with adhesive, and incubated at 25 °C to establish the G0 generation. G1-positive silkworm individuals with red eye fluorescence were mated with positive silkworms carrying the tissue-specific Ser1-Cas9 promoter (green eye fluorescence) to create the knockout strain, denoted as Ser^p^-*BmFADD*-KO.

### Western blotting and Co-IP assay

The harvested cells were lysed on ice for 30 min using a cell lysis buffer (Beyotime) containing phenylmethylsulfonyl fluoride (Beyotime) and phosphatase inhibitor (TransGen Biotech, Beijing, China), 72 h posttransfection. Subsequently, the lysates were centrifuged at 13,000 × g for 10 min for clarification, and the supernatant was retrieved. BmFADD antibody (1:100), Flag antibody (1:100; Abmart), and immunoglobulin G antibody (1:100; Abmart) were added and incubated overnight at 4 °C on a rotating instrument. Protein A/G magnetic beads were added to facilitate antibody binding, and the eluate was collected. Eluates were combined with sodium dodecyl sulfate (SDS) loading buffer and denatured by boiling at 100 °C for 10 min prior to resolution via SDS–polyacrylamide gel electrophoresis. The proteins were blotted onto polyvinylidene difluoride membranes (Roche, Basel, Switzerland) and subsequently probed with the following primary antibodies: anti-Flag (1:2,000; Abmart), anti-Myc (1:2,000; Abmart), anti-HA (1:2,000; Bioworld), anti-mTOR (1:1,000; CST, MA, USA), anti-p-4E-BP1 (1:1,000; CST), anti-p-S6K (1:1,000; CST), and anti-pan phospho-serine/threonine (1:2,000; ABclonal, Wuhan, China), followed by incubation with a secondary antibody (1:5,000; Beyotime). The signal was developed using western ECL luminescent solution (Yeasen Biotechnology).

### Y2H system

The bait and prey genes were inserted into the pGBKT7-BD and pGADT7-AD vectors, respectively, with the corresponding primers listed in Table S1. Recombinant BD and AD vectors were cotransformed into yeast competent cells according to the kit protocol (Coolaber, Beijing, China) and evenly spread on SD/-Leu/-Trp plates. Single colonies were selected, diluted 100-fold, and spotted onto SD/-Leu/-Trp/-His and SD/-Leu/-Trp/-His/-Ade plates to assess the interaction between the bait and prey.

### Isolation of nuclear proteins and cytoplasmic proteins

At 72 h posttransfection, cells were washed with phosphate-buffered saline and centrifuged to collect the pellet. Following the nuclear and cytoplasmic protein extraction kit instructions (Beyotime), cytoplasmic extraction buffer was added to the pellet to obtain the cytoplasmic fraction. After removing the supernatant, nuclear extraction buffer was added to the remaining pellet to isolate nuclear proteins. The entire extraction was performed on ice. Anti-tubulin (Beyotime) and anti-histone H3 (Beyotime) were used as internal controls for cytoplasmic and nuclear proteins, respectively.

### Image processing and statistical analysis

The GraphPad Prism software was used for data analysis, and Adobe Illustrator (version 2022) was used for graphic layout. Three biological replicates were set up for all experiments. Significant differences among all treatment groups were assessed via a Student *t* test. ***P < 0.001; **P < 0.01; *P < 0.05; ns, not significant.

## Ethical Approval

This article does not contain any studies with human or vertebrate animal subjects.

## Data Availability

The data that support the findings of this study are available from the corresponding authors upon reasonable request.
