# Supplementary material for: CDK2-Mediated Phosphorylation of Bombyx mori FADD Regulates S-Phase Progression and DNA Replication
Source: Research (Wash D C). 2026 Sep 22;9:1441. doi: 10.34133/research.1441 (PMC13594422; doi:10.34133/research.1441)
Supplement: Supplementary file 1 [file research.1441.f1.docx]

**Supplemental Materials**

Figures S1 to S7

**Figure S1. Characterization of *BmFADD* gene sequences and assessment of cell cycle synchronization.** (A) Analysis of the structure of *BmFADD* gene. (B) Prediction of the domain of BmFADD protein. (C) CCK-8 assay of cell viability after synchronization reagent treatment. (D) qRT-PCR analysis of *BmCyclin E* expression in different cell cycle-enriched populations.

**Figure S2.** **Construction of the expression vector of *BmFADD* gene.** (A) Western blot assay of the overexpression of BmFADD. (B) qRT-PCR detection of the overexpression level of *BmFADD* gene. (C) Detection of the knockout efficiency of *BmFADD* gene. D) qRT-PCR detection of the knockout level of *BmFADD* gene. (E) Flow cytometric analysis of the effects of *BmFADD* on cell cycle progression in total cell populations at 48h-post transfection. Data were presented as mean ± SD of three independent biological replicates. ****p* < 0.001; ***p* < 0.01; **p* < 0.05; paired Student’s *t*-test.

**Figure S3.** **Analysis of protein kinases that phosphorylate BmFADD.** (A) GO analysis of protein kinases based on LC-MS/MS identification results. (B) Prediction of the protein kinase at BmFADD Ser130 in iGPS 1.0. (C) Analysis of the interference efficiency of siRNAs. (D) Co-localization analysis between BmFADD and BmCDK1. (E) Analysis of the co-localization distance (pixel) between red and green fluorescence using Image J. Pearson’s correlation coefficient =0.981. (F) Co-IP validation of the interaction of BmFADD and BmCDK1. (G) Analysis of the co-localization distance (pixel) between red and green fluorescence using Image J. Pearson’s correlation coefficient =0.977. (H) Co-localization analysis of BmFADD and BmAurB. (I) Co-IP validation of the interaction between BmFADD and BmAurB. (J and K) Co-IP analysis of the phosphorylation level between BmFADD S130A mutant and BmFADD wild type. Data were presented as mean ± SD of three independent biological replicates. ***p* < 0.01; **p* < 0.05; paired Student’s *t*-test.

**Figure S4.** **Analysis of the stability of BmFADD protein.** (A) Immunofluorescence analysis of the expression efficiency of plasmids; scale bar = 5 μm. (B) Statistical analysis of the expression efficiency of plasmids. (C) qRT-PCR analysis of *BmFADD* expression after transfection with plasmids. (D-E) Analysis of cell proliferation following exposure to inhibitors of varying concentrations. (F) Cycloheximide chase analysis of the degradation of BmFADD-WT, BmFADD-S130A, and BmFADD-S130E. Data were presented as mean ± SD of three independent biological replicates. ****p* < 0.001; ***p* < 0.01; **p* < 0.05; ns, not significant, paired Student’s *t*-test.

**Figure S5.** **Analysis of protein subcellular localization.** (A) Nuclear localization signal prediction via NLStradamus; threshold marked 0.6. (B) Analysis of dephosphorylation mutations' influence on the subcellular localization of BmFADD; scale bar = 5 μm.

**Figure S6.** **Regulatory mechanism analysis of BmFADD and BmCDK2.** (A) Flow cytometric analysis of the effects of Ser130 phosphorylation and dephosphorylation on cell cycle progression in total cell populations at 48h-post transfection. (B) qRT-PCR analysis of the expression levels of *BmCDK2*, *BmCyclinE* and *BmCyclinA* after *BmFADD* overexpression and *BmFADD* knockout. (C–F) Western blot analysis of the effect of BmFADD overexpression and knockout on the expression of BmCyclinE. (G–H) Co-immunoprecipitation detection the interaction between BmFADD and BmCyclin E or BmCyclin A. (I–K) qRT-PCR analysis of *BmFADD* and *BmCDK2* expressions. (L–M) Co-immunoprecipitation detection the interaction capacity among BmFADD Ser130 mutants, BmCDK2 and BmCyclin E. Data were presented as mean ± SD of three independent biological replicates. ****p* < 0.001; ***p* < 0.01; **p* < 0.05; ns, not significant, paired Student’s *t*-test.

**Figure S7. Insert site and** **proteomic analysis of Ser^p^-*BmFADD*-KO strains.** (A) Schematic representation of the insertion site. (B) qRT-PCR analysis of the expression the insertion site genes. (C) Detection of knockdown efficiency in silk gland tissue of Ser^p^-*BmFADD*-KO strains. (D) Volcano plot of differentially expressed proteins in the fifth day of the fifth instar (5L5D) silk gland tissue, based on a fold change > 1.2 and *p*-value < 0.05. (E) EggNOG Enrichment analysis of differentially expressed proteins. Data were presented as mean ± SD of three independent biological replicates. ns, not significant, paired Student’s *t*-test.

Table S1

**Table S1. List of Primers.**

| **Primer Name** | **Primer Sequence (**5'–3'**)** |
| --- | --- |
| **Qsw22934** | F: TTCGTACTGGCTCTTCTCGT  R: CAAAGTTGATAGCAATTCCCT |
| **QBmCDK1** | F: AGGGCTCCTGAGGTCTTACT  R: TGTTGGCGTTCTTAGCATTCT |
| **QBmCDK2** | F: CTGAAGCCCCAGAACCTACTAATA  R: CGCACAGGGATACCAAAAGC |
| **QBmCyclin E** | F: TCCAAGCCCTACACCTCCAA  R: GCAGTCCTCATTTAGCCAGTCC |
| **QBmCyclin A** | F: CTGACAAACGGAACTTGTAAGG  R: GTGGGTTTGTCTTCATATACGC |
| **QBmCyclin B** | F: TGTCAAAAATGTTATTCAGCC  R: TTTCCGTAAAGAGTCAGTTCC |
| **QBmAurB** | F: GGTCAGTCCATGCTCCATCT  R: TGGAGGCTTTCCCACTAGAA |
| **QBmFADD** | F: GTACAAACTGGCGGGCTTTA  R: ATCGCGCCTGTATGATTCTT |
| **QBmTOR** | F: ACCGGGCTCTATGAAAACCT  R: TGAGCACGTTCAGAAACTGG |
| **BmCDK2-Myc** | F:CGGGATCCATGGAGCAGAAACTCATCTCTGAAGAGGATCTGGAGAATTTTTCTACTGTAGAAA  R:GCTCTAGATTACAGATCCTCTTCAGAGATGAGTTTCTGCTCAGGATGTAGAGGAGGCGGCACC |
| **BmCDK1-HA** | F:ATTTGCGGCCGCATGTACCCATACGATGTTCCAGATTACGCTGATGATTTCTTAAAGAT  R:GCTCTAGATTAAGCGTAATCTGGAACATCGTATGGGTATACACTTTGAACAGAATCCGT |
| **BmAurB-HA** | F:GGGGTACCATGTACCCATACGATGTTCCAGATTACGCTACAATGAAGAGCGAAGTGCTC  R:GGACTAGTCTAAGCGTAATCTGGAACATCGTATGGGTATACTGTCTGAAATTGTTGGAC |
| **BmFADD-(1-68aa)-Flag** | F:GGGGTACCATGGATTACAAGGATGACGACGATAAGACTAAGTCAGAATATTCACT  R:GGACTAGTTTACTTATCGTCGTCATCCTTGTAATCAATGCTATTTTCAGACAATACATCT |
| **BmFADD-(69-135aa)-Flag** | F:GGGGTACCATGGATTACAAGGATGACGACGATAAGGAGCCACTGAAAGAAGTCGCAC  R:GGACTAGTTTACTTATCGTCGTCATCCTTGTAATCTTCTTTTTTTCTTGGACTTAAA |
| **BmFADD-(136-221aa)-Flag** | F:GGGGTACCATGGATTACAAGGATGACGACGATAAGAGACTATTTGAAACTATAATAG  R:GGACTAGTTTACTTATCGTCGTCATCCTTGTAATCAATATTCATTACTATAATATTT |
| **BmFADD-sgRNA** | F: AAGTGGCAAGTGGAACTCCAAGACA  R: AAACTGTCTTGGAGTTCCACTTGCC |
| **BmFADD -Flag** | F:GGGGTACCATGGATTACAAGGATGACGACGATAAGACTAAGTCAGAATATTCACT  R:GGACTAGTTTACTTATCGTCGTCATCCTTGTAATCAATATTCATTACTATAATATTT |
| **U6-Bgl Ⅱ** | F: GGAAGAATCTTCCAGGTTATGTAGTACACATT  R: GGAAGATCTTCCAAAAAAAGCACCGACTCG |
| **BmCyclin E-HA** | F:GGGGTACCATGTACCCATACGATGTTCCAGATTACGCTGCTGAATCTGGGTCTCCC  R:GGACTAGTTCAAGCGTAATCTGGAACATCGTATGGGTAGTCAGCATGTATCCTTAG |
| **BmCyclin A-HA** | F:GGGGTACCATGTACCCATACGATGTTCCAGATTACGCTGCTTTTAGAATTCATGAA  R:GGACTAGTCTAAGCGTAATCTGGAACATCGTATGGGTAGCTGGTGGCGCGCGCAGA |
